# Supplementary material for: End Sequence Analysis Toolkit (ESAT) expands the extractable information from single-cell RNA-seq data
Source: Genome Res. 2016 Oct;26(10):1397–410. doi: 10.1101/gr.207902.116 (PMC5052061; doi:10.1101/gr.207902.116)
Supplement: Supplemental Material [file supp_gr.207902.116_Supp_Materials.tar.gz › ESAT-esat_v0.1/doc/html/classjaligner_1_1_traceback_node.html]

ESAT: jaligner.TracebackNode Class Reference


ESAT


- Main Page
- Related Pages
- Packages
- Classes
- Files

- Class List
- Class Index
- Class Hierarchy
- Class Members

All Classes Namespaces Files Functions Variables Enumerator Pages

- jaligner
- TracebackNode

Public Member Functions |
List of all members

jaligner.TracebackNode Class Reference

|  |  |
| --- | --- |
| Public Member Functions | |
|  | TracebackNode () |
|  | |
| void | setDiagonal () |
|  | |
| void | setUp (int length) |
|  | |
| void | setLeft (int length) |
|  | |
| void | setStop () |
|  | |
| byte | getDirection () |
|  | |
| int | getLength () |
|  | |

## Detailed Description

Traceback node

Author
:   Bram Minnaert
:   Ahmed Moustafa

## Constructor & Destructor Documentation

|  |  |  |  |  |
| --- | --- | --- | --- | --- |
| jaligner.TracebackNode.TracebackNode | ( |  | ) |  |

Constructor

## Member Function Documentation

|  |  |  |  |  |
| --- | --- | --- | --- | --- |
| byte jaligner.TracebackNode.getDirection | ( |  | ) |  |

Returns
:   Returns the direction.

|  |  |  |  |  |
| --- | --- | --- | --- | --- |
| int jaligner.TracebackNode.getLength | ( |  | ) |  |

Returns
:   Returns the length.

|  |  |  |  |  |
| --- | --- | --- | --- | --- |
| void jaligner.TracebackNode.setDiagonal | ( |  | ) |  |

Sets the direction to diagonal

|  |  |  |  |  |  |
| --- | --- | --- | --- | --- | --- |
| void jaligner.TracebackNode.setLeft | ( | int | *length* | ) |  |

Sets the direction to left

Parameters
:   |  |  |
    | --- | --- |
    | length | length of gap |

|  |  |  |  |  |
| --- | --- | --- | --- | --- |
| void jaligner.TracebackNode.setStop | ( |  | ) |  |

Sets the direction to stop

|  |  |  |  |  |  |
| --- | --- | --- | --- | --- | --- |
| void jaligner.TracebackNode.setUp | ( | int | *length* | ) |  |

Sets the direction to up

Parameters
:   |  |  |
    | --- | --- |
    | length | length of gap |

---

The documentation for this class was generated from the following file:

- java/jaligner/TracebackNode.java


---

Generated on Fri Aug 15 2014 15:11:05 for ESAT by  

 1.8.7
